# A Study on the Improvement of the Durability of an Energy Harvesting Device with a Mechanical Stopper and a Performance Evaluation for Its Application in Trains

**DOI:** 10.3390/mi11090785

**Published:** 2020-08-19

**Authors:** Jaehoon Kim

**Affiliations:** Department of Advanced Railroad Vehicle Research, Korea Railroad Research Institute, Uiwang 16105, Korea; lapin95@krri.re.kr

**Keywords:** energy harvesting, durability, design, generated power, train, wireless sensor

## Abstract

Durability is one of the critical issues concerning energy harvesting devices. Even with the energy harvesting device’s excellent performance design, the moving components, such as the spring, get damaged during operation. In this study, an energy harvesting device was designed for durability improvement. The mechanical stopper of the energy harvesting device was selected as a new design component to prevent spring damage. An experimental and finite element analysis (FEA) was carried out on the amount of energy harvesting power possible using a mechanical stopper to improve the durability of the energy harvesting device. A performance evaluation of the energy harvesting device using the mechanical stopper was conducted under laboratory and driving conditions of a high-speed train traveling at 300 km/h. The measurement of the generated power gives the target value for the minimum performance of the newly designed energy harvesting device used as the power source of the wireless sensor node for high-speed trains.

## 1. Introduction

In recent years, due to human-caused accidents and natural disasters, the demand for an improved reliability and safety for systems and structures has increased, and the increase in the maintenance work for these systems and structures has emerged as another problem [1]. Therefore, new technology development efforts are needed to address both aspects—“improving reliability and safety” and “reducing maintenance costs”. In order to do this, the system and structure must be intelligent. The basis of such intelligence is the development of a continuous detection technology. For this, intelligent monitoring with sensors and wireless communication technology, such as a wireless sensor node, are required [2].

Technological advancements have reduced the power consumption of the wireless sensor node and have facilitated new applications in intelligent monitoring for systems and structures. However, the need for a continuous power supply remains a challenge [3]. The requirements for an extended period of power for wireless sensor nodes have brought about the need for alternative energy sources. Energy harvesting technology is a possible method to increase the power supply by utilizing an existing environmental energy source [4,5]. Among the various environmental energy sources, vibration energy exists in various structures and attracts attention as an energy source that could be harvested, thereby supplying power to wireless sensors [6,7]. Energy harvesting from vibration energy has been applied to various types of wireless sensors that can be used to monitor the condition of systems and structures such as bridges [8,9,10,11], pipelines [12], and wind turbines [13,14].

The same concept is apparent in the systems of trains [15]. The high-speed operation of trains makes reliability and safety of great importance, and consequently, the cost of train maintenance has steadily increased. Thus, intelligent monitoring with a wireless sensor node is needed for the early detection of abnormal conditions to mitigate system failures and accidents [16,17,18]. Trains currently use wired sensors mostly [19]. Recently, the demand for an intelligent monitoring system using a wireless sensor node has been gradually increasing; however, there are limitations at present regarding the installation and the difficulty of accessing trains. Some significant benefits must be taken into account. In particular, when applying the intelligent monitoring system to the train using a wireless sensor node, maintenance based on the real driving conditions while moving becomes possible, which differs from the methods currently used in maintenance management, such as periodic disassembly and inspections. As a result, the reliability and stability of the train can be improved [20].

However, even in the case of a wireless sensor node with fewer installation and location constraints, the power supply problem must be solved for the intelligent monitoring system. As the battery’s current energy density rate does not meet the application’s demands, periodic battery replacement is necessary for the real-time or long-term intelligent monitoring of a train system. The battery, which consumes energy continuously, is a limited resource, not environmentally friendly, and generates additional maintenance tasks [21]. Therefore, the intelligent monitoring of train systems requires the development of an environmentally friendly and semi-permanent “energy harvesting” technology exploiting the ambient energy generated during system operations [20,22,23,24,25]. The energy harvesting device’s research using electromagnetic induction should analyze the mechanical motion [22,26,27,28,29]. The mechanical motion characteristics can be classified into vibration and rotational motion [16,23,24,25,30,31,32].

The electromagnetic vibration energy harvesting (forward, VEH) device of an intelligent monitoring system on a train using a wireless sensor node holds a great deal of promise. The previous study verified the applicability of the energy harvesting technology that produces electric energy from a vibration energy source associated with the high-speed train operation. An analytic estimation study was carried out on the amount of energy harvested from the high-speed train’s vibrational acceleration based on the theoretical model [1]. The design study was carried out on the amount of energy harvesting power possible, considering the VEH device’s design factors. A new design of the VEH device was suggested based on the results [5]. However, durability is one of the critical issues concerning the VEH device. Even with the VEH device’s excellent performance design, the moving components were damaged during operation. The energy harvesting device’s durability research was conducted to consider the aspect of design and materials [33,34,35]. In this study, the VEH device was designed for durability improvement. The mechanical stopper of the VEH device was selected as a new design component to prevent spring damage. An experimental and finite element analysis (FEA) was carried out on the possible amount of energy harvesting power using the mechanical stopper to improve the durability of the VEH device. The characteristics of the generated power were studied using a mechanical stopper in the laboratory and on a high-speed train. The measurement of the generated power gives a target value for the minimum performance of the newly designed practical VEH device to be used as a power source for the wireless sensor node for the high-speed train.

## 2. The Performance of the VEH Device and the Durability Problem

A design study for a resonant VEH device for the train was conducted. The VEH device requires an analysis using the equation of motion: the mass(m)-spring(k)-damper(d) system [36,37]. When the VEH device is vibrated, the mass moves out of phase with the VEH device frame, so there is net movement between the mass and the frame. This relative displacement is sinusoidal in amplitude and can drive a suitable transducer, thereby generating electrical energy. The transducer is depicted as a dashpot because the conversion of mechanical energy into electrical energy dampens the vibration. In sinusoidal vibrations, the analytical power dissipated by a mass-spring-damper system when its base is vibrating at its natural frequency can be calculated as follows [1,37]:

(1) $$P_{T}=\frac{mA^{2}}{4\omega_{n}\xi_{T}}$$

where:

*P*_T_ is the total dissipated power (W)
*m* is the vibrating mass (kg)
*A* is the vibration acceleration amplitude of the frame of the mass-spring-damper system (m/s^2^)
*ω*_n_ is the undamped natural frequency of the mass(m)-spring(k)-damper(d) system (rad/s)
$\xi_{T}$ is the total damping ratio (−).

The vibration excitation of the mass-spring-damper-system based on the VEH device can be spread over a wider frequency band in the context of a train. The dependency between the movement of the mass–spring–damper system frame and the relative movement of the mass concerning the frame can be modeled by the following differential equation [38]:

(2) $$m\ddot{z}\left(t\right)+d_{T}\dot{z}\left(t\right)+kz\left(t\right)=-m\ddot{y}\left(t\right)$$

where:

*z*(*t*) is the relative position of the mass concerning the frame (m)
*y*(*t*) is the position of the frame (m)
*d*_T_ is the total damping constant (kg/s)
*k* is the spring constant (N/m = kg/s^2^).

The previous study verifies the applicability of VEH devices in producing electric energy from a vibration energy source, according to the operation of a high-speed train, for an intelligent monitoring system on a high-speed train using a wireless sensor node [1]. An analytic estimation study was carried out on the amount of energy harvested from the vibrational acceleration of a high-speed train based on the theoretical model of Equations (1) and (2). The estimated harvestable power from the axle vibrations varied between 65.96 and 305.58 mW for the high-speed train. However, the available energy is assumed to be 70% of the generated power [39]. Therefore, the VEH device, based on the analytic estimation results, was set to generate over 50 mW of the minimum target power. It is slightly higher than the 70% of the minimum estimation: 46.17 mW, at the resonant frequency with a low acceleration, 0.5 G.

**Figure 1:**
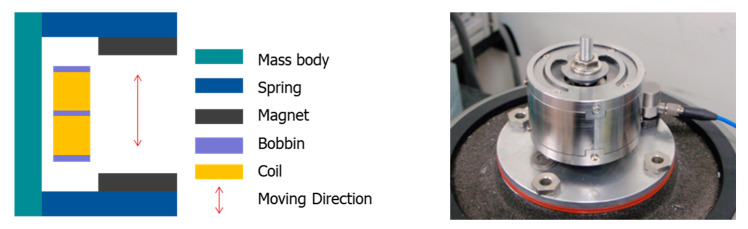
The schematic of the VEH device structure and the VEH device for the test.

It is necessary to design a VEH device which has a smaller size and a practical power generation performance for applications on trains. To increase the generated power and miniaturize the VEH device, a new design was needed to increase the magnetic field size, which is a significant factor in power generation performance when applied to the train. The VEH device was designed so that the same magnets (neodymium, Nd) were arranged in double, and two coils were also arranged similarly to correspond to each upper and lower magnet, as shown in Figure 1 [5]. This structural design considered changing the magnet’s size while reducing the internal height, securing the maximum moving space (spring displacement) of the coil due to vibration. Each coil of a VEH device can be connected in series or parallel. The steel material was applied to the upper and lower parts by applying plating to the spring structure using the method widely used in the structural design of accelerometers and gyro sensors—electromagnetic induction. The VEH was designed to enable the changing of the resonant frequency by using the thickness of the spring and the intermediate-mass body, so that the power generation can be maximized at the resonant frequency. The magnets were the moving component of the VEH device and moved in the direction of motion.

Many experimental studies can be found in the literature and were carried out on the amount of energy harvesting power based on the design factors of energy harvesting devices to verify the applicability of the energy harvesting technology that produces electrical energy from a vibrational energy source under testbed conditions [40,41,42,43]. The VEH devices had been tested for their power generation performance in laboratories and high-speed train field tests. The root mean square (forward, RMS) value of the generated voltage (V_rms_) analysis was performed for each load resistance (R). The experimental average generated power (P_exp_) was calculated using Equation (3), with the RMS value of the generated voltage (V_rms_) and the load resistance (R) [44]. The RMS generated voltage (V_rms_) is used to calculate the average power the harvesting device delivered to the load (i.e., the power dissipated across the load resistance).

(3) $$P_{\exp}=\frac{{\left(V_{\mathrm{rms}}\right)}^{2}}{R}$$

**Figure 2:**
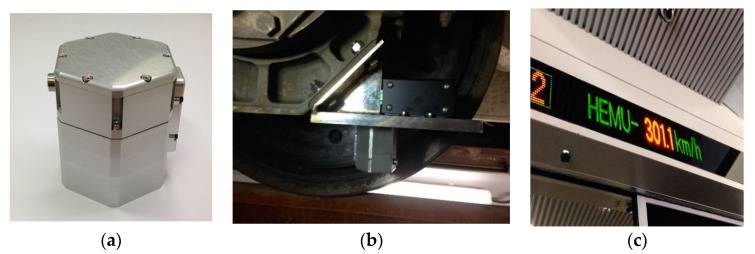
The VEH device with the protecting case (**a**), the VEH device for the high-speed train test on the axle box (**b**), the test speed example of the high-speed train during the VEH device test: 301.1 km/h (**c**).

The evaluation of the power generation performance of the VEH device was conducted under controlled conditions in the laboratory and on the high-speed train in the previous study [5]. The maximum values of the generated power (P_exp-veh_) in the VEH device were 265.7 and 765.4 mW at 0.5 to 2.0 G of vibration acceleration, respectively, in the laboratory test. The test results confirmed that the VEH device has a relatively low Q factor, independent of the resonant frequency. The same VEH device, which was confirmed through laboratory tests, was installed on the axle box of the high-speed train to verify its performance. Figure 2 shows the installed VEH device on the test jig of the axle box of the high-speed train. The maximum value of V_rms-veh-T_ was confirmed to be 13.91 V, and the RMS generated voltage with a 360 Ω load resistance (V_rms-veh-T_) was calculated as 5.16 V for 2000 s of the total time interval at one single coil in the high-speed train test. In this case, a 360 Ω load resistance was similar to the 368 Ω internal resistance of one coil in the VEH device. The total values of the experimentally RMS generated power with a 360 Ω load resistance (P_exp-veh-T_) of one VEH device, which was in serial connection to two coils, was 147.92 mW on the high-speed train test, which was traveling at a maximum speed of 301.1 km/h over a distance of 130.7 km.

**Figure 3:**
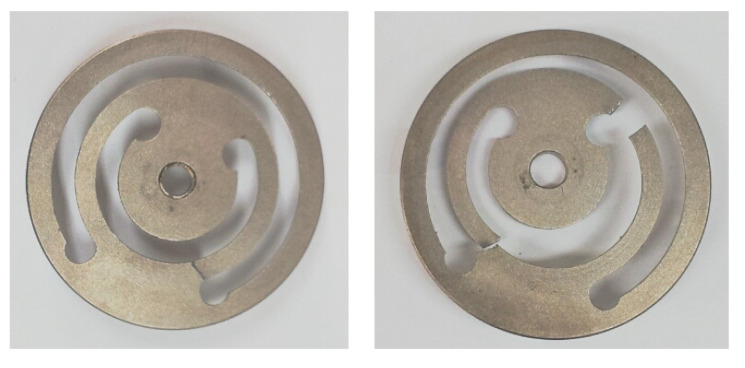
The broken springs of the VEH device during the high-speed train test.

However, durability is one of the critical issues in the designing of a VEH device. Even with the VEH device’s excellent performance design, the moving components, such as the spring, were damaged during the operation. In this case, the springs of the VEH device were broken due to the high impact of the vibration acceleration during the variable operation conditions (velocity, wheel-rail condition, etc.), as shown in Figure 3.

**Figure 4:**
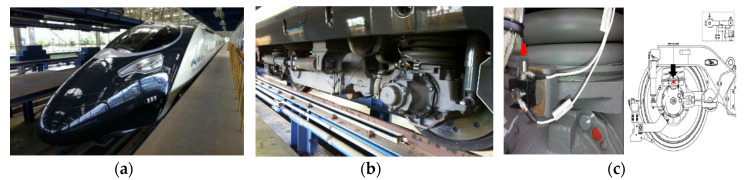
The sensor installation position on the axle of a high-speed train: (**a**) the high-speed train, (**b**) the bogie of the high-speed train, (**c**) the sensor at the axle box.

**Figure 5:**
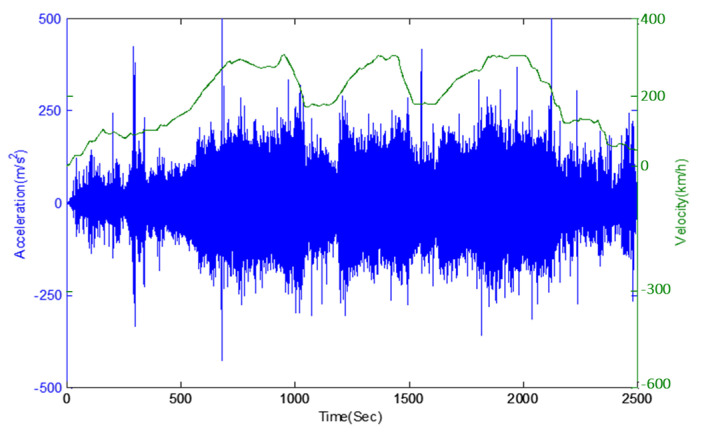
The vibration acceleration data on the axle box with velocity (km/h): (blue line) the vibration acceleration, (green line) the velocity data during the driving at 300 km/h. The maximum impact of the vibration acceleration occurred between about ± 30 (±300 m/s^2^) and about ± 50 G (±500 m/s^2^) during the period when the high-speed train traveled at 300 km/h.

In this study, the vibration acceleration was measured on the high-speed train to find how much the vibration acceleration impacted the VEH device’s ability to evaluate the durability of the VEH device on the high-speed train under real driving conditions. As shown in Figure 4, the acceleration sensor, with a range of ±50 G, was installed on the axle box of the high-speed train to measure the vibration acceleration at the axle (sampling rate: 1 kHz). The maximum impact of the vibration acceleration on the axle occurred between about ± 30 (300 m/s^2^) and ± 50 G (500 m/s^2^) during the period when the high-speed train traveled at 300 km/h, as shown in Figure 5.

**Figure 6:**
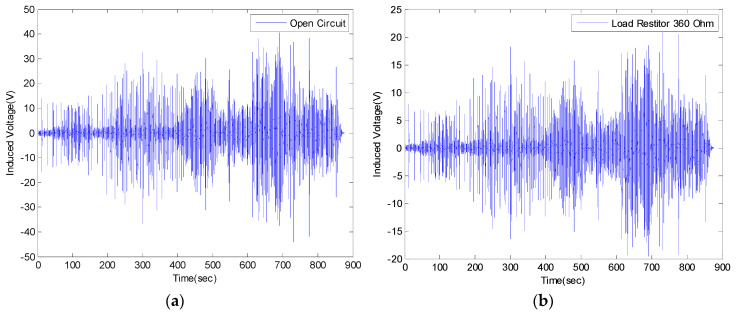
The generated voltage of the VEH device for the high-speed train for the lower coil before the spring damage on the high-speed train test about 900 s: (**a**) the induced voltage (V); open-circuit peak to peak voltage and (**b**) the induced voltage (V) with a 360 Ω load resistance—the peak to peak voltage.

**Figure 7:**
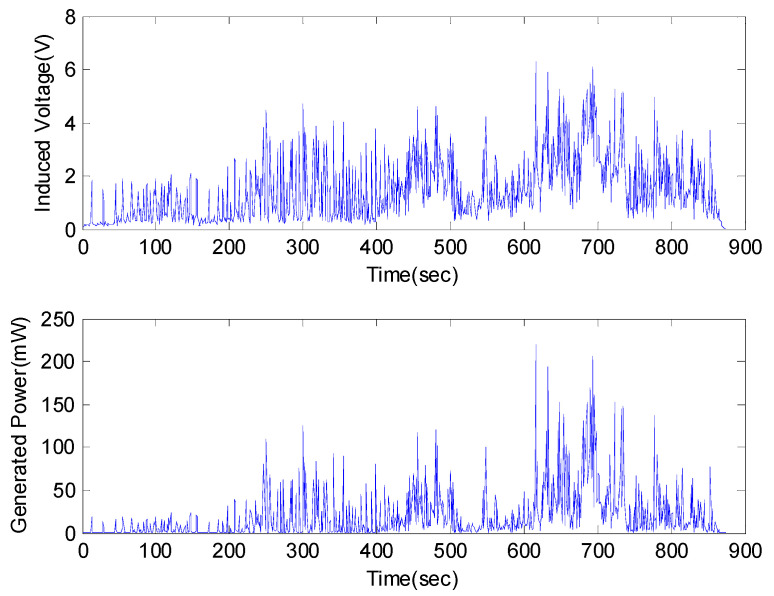
The root mean square (forward, RMS) generated voltage with a 360 Ω load resistance (V_rms-veh-T_) (y-axis) (**top**) and the experimentally generated power (P_exp-veh-T_) (y-axis) (**bottom**) by every 1-s time interval of the VEH device on the high-speed train at one lower coil before the spring damage in the high-speed train test was about 900 s: the RMS V_rms-veh-T_ was 1.87 V and the P_exp-veh-T_ was 9.71 mW (one lower coil).

The results of the real-time generated voltage of the VEH device while the high-speed train was driving are shown in Figure 6 and Figure 7. The results were expressed in terms of a one lower coil basis in the VEH device for the standardization of the test results. Each coil of the VEH device can be connected in series or in parallel. The spring of the VEH device was affected by the high impact of the vibration acceleration, which was between ± 30 and ± 50 G. The spring was broken during the actual driving test before 900 s as shown in Figure 6. During this short time, the maximum values of the peak to peak voltage in both the open-circuit case and 360 Ω load resistance case were measured between ± 40 and ± 20 V at the VEH device. The RMS generated voltage, with a 360 Ω load resistance (V_rms-veh-T_), was calculated as 1.87 V before the spring broke, as shown in Figure 7. In this case, a 360 Ω load resistance was similar to the 368 Ω internal resistance of one coil in the VEH device. Therefore, as the result of the V_rms-veh-T_ of the VEH device and Equation (3), the experimentally generated power with a 360 Ω load resistance (P_exp-veh-T_) was calculated to be 9.71 mW for one coil of the VEH device. The total P_exp-veh-T_ of the VEH device connected in serial to the upper coil and the lower coil was 19.42 mW before the spring failed in the high-speed train test. These values of the RMS generated voltage (V_rms-veh-T_) and generated power (P_exp-veh-T_), before the spring failure, were significantly lower than the previous condition of the VEH device.

## 3. The Durability Improvement for the VEH Device

### 3.1. The Design Study of the Durability Improvement for the VEH Device, and the Laboratory Verification Test

As the above results show, durability is an essential issue for VEH devices. Since VEH devices use the incoming vibration acceleration load, the vibration acceleration is an energy source and an external force that influences the durability simultaneously. As shown in Figure 5 for the high-speed train, the impact of the vibration acceleration occurs from ± 30 to ± 50 G on the axle. Therefore, the spring’s metal material (of the VEH device) does not easily have durability when high vibration accelerations are inputted in a form such as an impact load and periodic. In this study, the design and experimental studies were carried out in order to determine the amount of energy harvesting power possible when considering a design that prevents spring damage to improve the durability of a VEH device for use on high-speed trains.

The mechanical stopper of the VEH device was the newly selected component to prevent spring damage. Only the mechanical stopper was changed from the original VEH device’s design structure for the laboratory and the high-speed train tests. The mechanical stopper, a ring-type of ethylene propylene diene monomer (forward, EPDM) rubber material (hardness shore A65), was applied. SKD 61 steel (Japan Industrial Standards hot die steel code, forward, SKD 61) was applied to the spring structure material; the resonant frequency of the VEH device was designed at 50 Hz. The magnets (neodymium (forward, Nd), size: outer diameter 25 mm, inner diameter 10 mm, thickness 7 mm) were arranged in double, and two coils were arranged in duplicate to correspond to the upper and lower magnet. The maximum moving displacement of the spring was 4.0 mm without the mechanical stopper in the VEH device, and the moving displacement of the spring with the mechanical stopper was determined in the following study.

**Figure 8:**
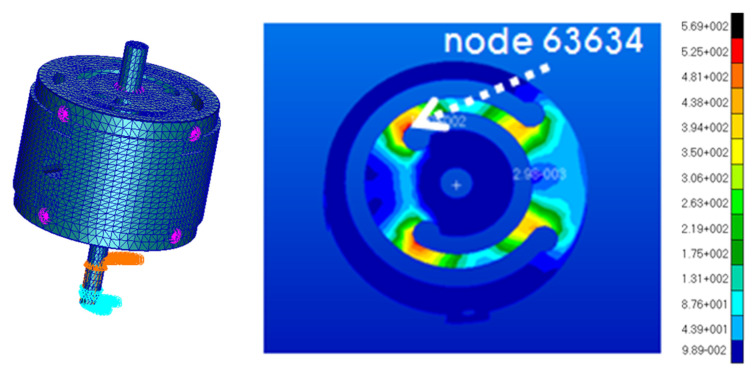
The finite element analysis (FEA) model of the VEH device and the stress distribution on the spring.

**Figure 9:**
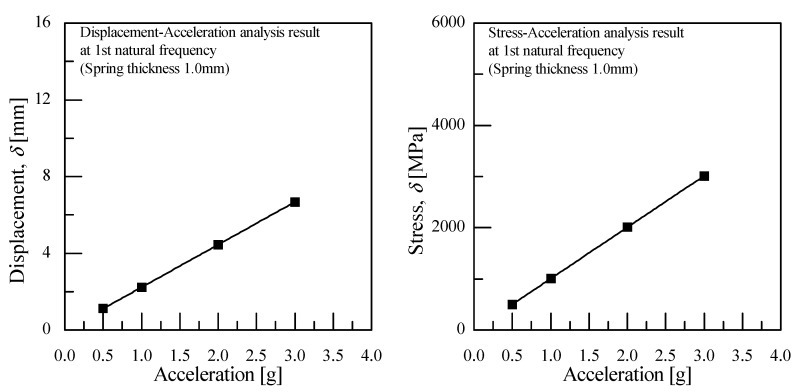
The results of the finite element analysis (FEA) for the displacement and the stress according to the vibration acceleration.

**Figure 10:**
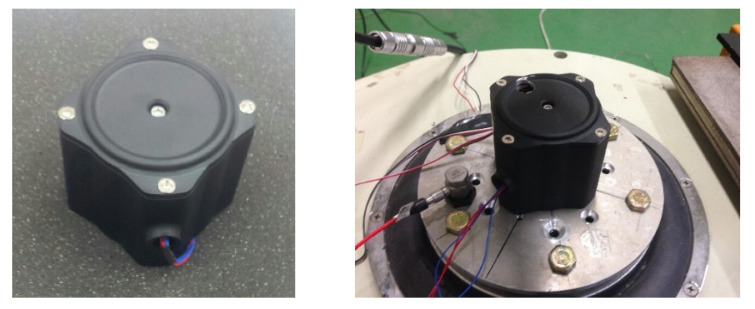
The VEH device and the vibrator it was on.

**Figure 11:**
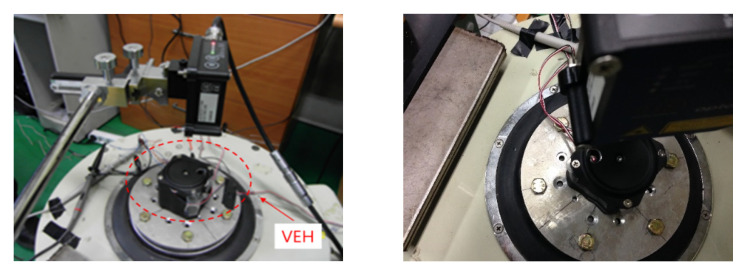
The test of the VEH device in the laboratory and the laser displacement meter for measuring the moving displacement.

**Figure 12:**
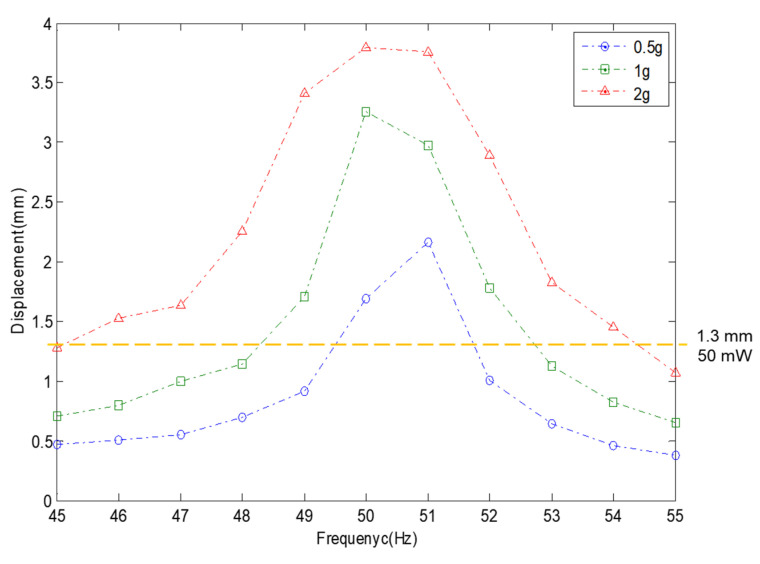
The test results of the moving displacement for the spring according to the vibration acceleration at one lower coil of the VEH device. The VEH device with a resonant frequency of 50 Hz was tested to increase the frequency by 1 Hz in the 50 Hz ± 5 Hz frequency band under 0.5, 1.0 and 2.0 G vibration acceleration conditions. The thickness of the spring was 1.0 mm. The maximum moving displacement of spring was 4.0 mm.

As shown in Figure 8, the finite element analysis (FEA) of the frequency responses for the VEH device was conducted before the laboratory test compared the maximum displacement and the maximum stress of the spring according to the vibration acceleration [45,46]. The finite element analysis (FEA) used solid (CTETRA) and shell (CTRIA3) elements with 49,064 elements, 14,507 nodes, and multi-point constraints MSC Patran/Nastran. The boundary conditions were applied to 0.5, 1.0, 2.0, and 3.0 for the vibration acceleration with MPC (multi-point constraints) at the solid and shell elements. The damping coefficient was set by the displacement measured in the performance test of the VEH device. The material properties of SKD 61 were 205.23 GPa (Young’s modulus); 0.29 (Poisson’s ratio); 9.19 × 10^3^ kg/m^3^ (density); 1955 MPa (tensile strength). The thickness of the spring was 1.0 mm and the resonant frequency was 50 Hz. As a result of the finite element analysis (FEA) in Figure 9, it was confirmed that the maximum stress occurred at node No. 63634, and the vibration test confirmed the same position of the spring failure as shown in Figure 3. The displacement and stress values increased linearly with an increasing input vibration acceleration. The displacement of about 4.0 mm was observed at a 2.0 G vibration acceleration. At this time, it was confirmed that the maximum stress value was very high, at about 2000 MPa, and it was 3000 MPa at 3.0 G. With such results, the mechanical spring of the VEH device did not easily have durability when high vibration accelerations are input at such an impact load. The experiment in the laboratory also confirmed a similar result of the finite element analysis (FEA) [45,46]. The evaluation of the moving displacement of the spring and generation performance of the VEH device was conducted under controlled conditions in the laboratory as shown in Figure 10 and Figure 11. The same VEH device was installed on the vibrator to verify its performance. Only the material of the protecting case was changed from steel to aluminum in the VEH device. The system of the laboratory test was comprised as follows: a vibrator, a data recorder, an accelerometer, and a laser displacement meter for measuring the moving displacement. A data acquisition board (DAQ) was used for the analog data and the software for analyzing the measured voltage data was MATLAB. The VEH device was tested with the frequency increasing by 1 Hz in the 50 Hz ± 5 Hz frequency band under the same 0.5, 1.0, 2.0 and 3.0 G vibration acceleration conditions in the finite element analysis (FEA), as shown in Figure 12. The results were expressed in terms of one lower coil basis in the VEH device as mentioned above.

**Figure 13:**
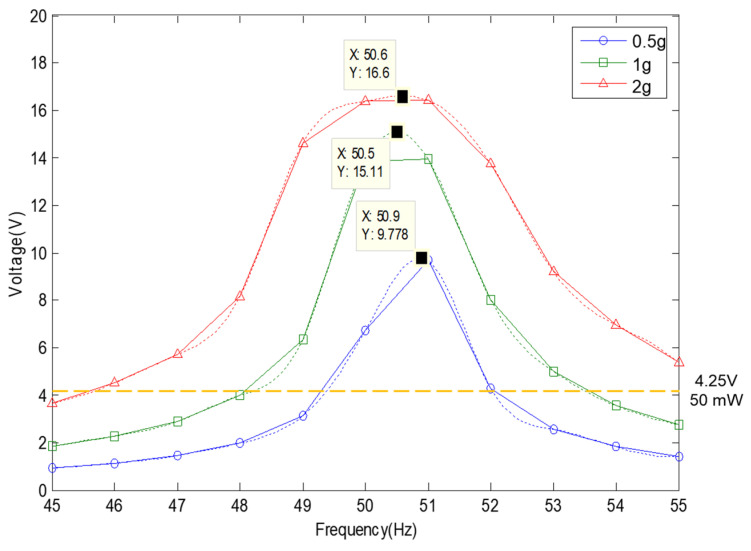
The RMS generated voltage with a 360 Ω load resistance (V_rms-veh_) according to the vibration acceleration at one lower coil of the VEH device. A resonant frequency of 50 Hz was used under 0.5, 1.0 and 2.0 G vibration acceleration conditions. The thickness of the spring was 1.0 mm. The maximum moving displacement of the spring was 4.0 mm.

**Figure 14:**
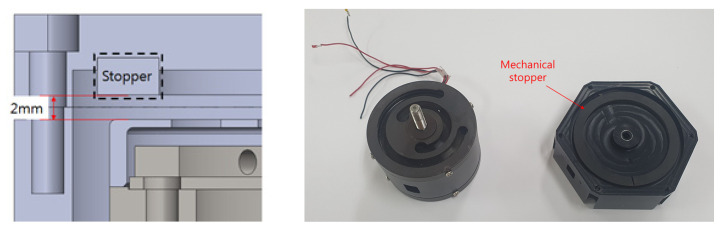
The maximum moving displacement of the spring was 2.0 mm with the mechanical stopper. As for the mechanical stopper, a ring-type of ethylene propylene diene monomers (EPDMs) ethylene rubber material was selected.

The value of the generated voltage (V_rms-veh_), in which the spring had about 4.0 mm of moving displacement, was 16.6 V with a 360 Ω load resistance at a 2.0 G vibration acceleration, as shown in Figure 12 and Figure 13. The spring was broken at the input of a 3.0 G vibration acceleration, and a performance test was not conducted. The maximum values of the P_exp-veh_ were calculated to be 265.7 mW to 765.4 mW at 0.5 G to 2.0 G of vibration acceleration, as shown in Table 1. Therefore, if the values of generated voltage were over 4.25 V, as shown in Figure 13, the values of the P_exp-veh_ in the VEH device will be over 50 mW, which is the minimum target performance of the VEH device for the system of the train. The minimum value of the moving displacement of the spring (from 0 mm to upside) was about 1.3 mm at 0.5 G to exceed the required voltage—4.25 V. Therefore, a complementary structural component to prevent damage to the mechanical spring and limit the range of moving displacement was applied through the use of a mechanical stopper. This also limits the excessive deformation, allowing up to 2 mm. The moving displacement of the spring was determined to be 2.0 mm with the mechanical stopper, in which the limit displacement was set to 2 mm, which gave a 50% margin for the minimum displacement of 1.3 mm of the target generation amount, as shown in Figure 14.

**Figure 15:**
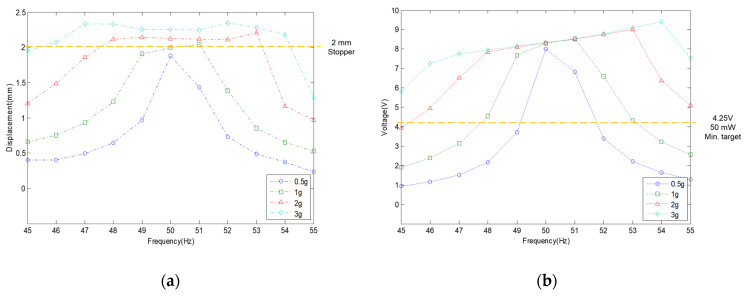
(**a**) The test results of the moving displacement for spring according to the vibration acceleration by the mechanical stopper, limited to 2 mm. (**b**) The RMS generated voltage with a 360 Ω load resistance (V_rms-veh_) according to the vibration acceleration at one lower coil of the VEH device: a resonant frequency of 50 Hz was used under 0.5, 1.0, 2.0, and 3.0 G vibration acceleration conditions; the thickness of the spring was 1.0 mm.

As a result of the above research, the evaluation of the moving displacement of the spring and the generation performance of the VEH device with the mechanical stopper was also conducted under the same test conditions of the VEH device without the mechanical stopper. To prevent damage to the mechanical springs, the moving displacement of the spring was reduced to 2.0 mm through the application of the mechanical stopper, thereby limiting deformation, and it confirmed that the mechanical stopper, a ring-type of EPDM rubber material, restricted the moving displacement from a 2.0 G vibration acceleration or higher, as shown in Figure 15.

The moving displacement of the spring at a low input vibration acceleration was limited to 2.0 mm, as shown in Figure 15a. As the vibration acceleration increased, the displacement increased to 2.3 mm at a 3.0 G vibration acceleration due to the rubber material’s elasticity. These displacement limits also reduce the generated voltage (V_rms-veh_), resulting in a value of the generated voltage (V_rms-veh_) of up to 9.41 V at a 3.0 G vibration acceleration, as seen in Figure 15b, at which the maximum value of the generated power (P_exp-veh_) was calculated at 245.97 mW, as shown in Table 2. However, the laboratory tests confirmed that a generated voltage (V_rms-veh_) higher than 4.2 V corresponds to the target generated power (P_exp-veh_) of 50 mW for the VEH device at a 50 Hz resonance frequency with the 2 mm displacement limit from more than a 0.5 G vibration acceleration.

**Figure 16:**
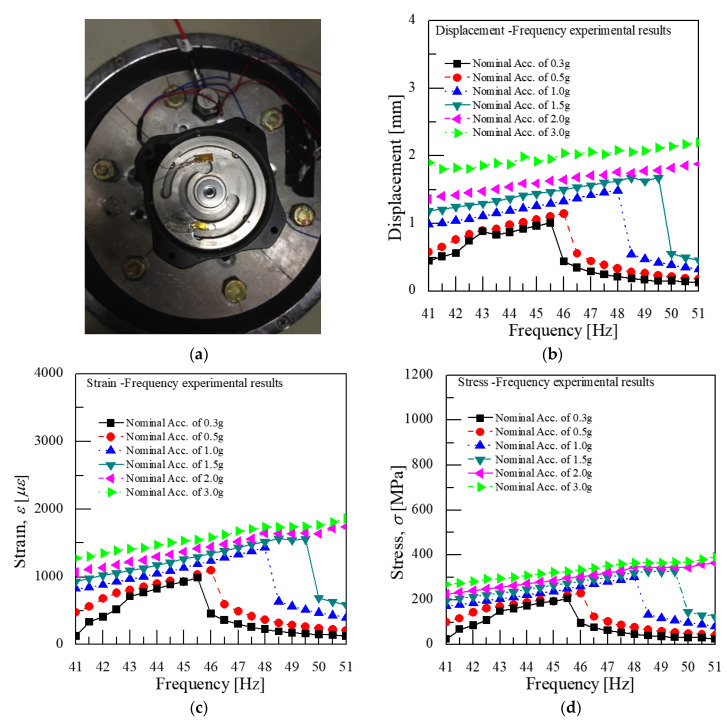
(**a**) The strain gauges on the spring of the VEH device at the stress–strain concentration place; (**b**) the change in both the displacement and the frequency according to the vibration acceleration; (**c**) the change in both the strain and the frequency; (**d**) the change in both the stress and the frequency. The thickness of the spring was 0.7 mm.

In addition, a study was conducted to find the effects of the moving displacement of springs and the stress changes according to the spring thickness under the limited moving displacement of spring. This experiment was performed by newly producing a 0.7 mm thickness of spring, lowering the resonant frequency of the VEH device to 45 Hz in a vibration acceleration range of 0.3 to 3.0 G, and comparing the results of the previous spring thickness of 1.0 mm and the resonant frequency that was set at 50 Hz. As shown in Figure 16, the moving displacement of the spring was limited to 2.0 mm above a 2.0 G vibration acceleration by applying the mechanical stopper similarly to the result of the 1.0 mm spring thickness. However, unlike the test results of the 1.0 mm spring in Figure 15a, the resonant frequency of a 0.7 mm spring thickness increased by 0.5 Hz from the original 45 Hz resonant frequency at a lower vibration acceleration input, 0.3 G, as shown in Figure 16b. It was reasoned that the vibration acceleration increased, the displacement of the spring increased more in a low thickness spring, and the spring’s stiffness increased, too. Therefore, the resonant frequency also increased more due to the use of a low thickness spring [47,48]. Thus, the resonance frequency was significantly changed by increasing the vibration acceleration, indicating a change of 3 Hz at a 1.0 G vibration acceleration and 5 Hz at a 1.5 G vibration acceleration at a more massive vibration acceleration.

**Figure 17:**
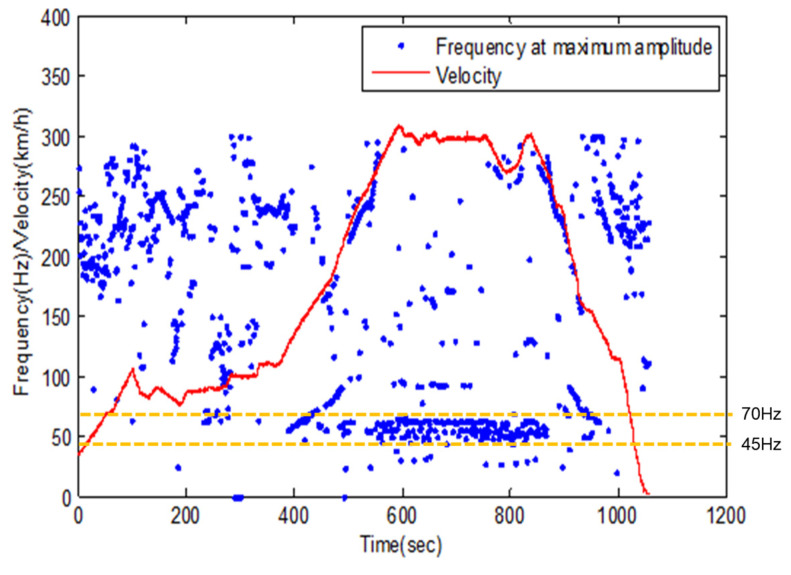
The fast fourier transform (FFT) results and velocity data, the resonant frequency occurred mainly when the frequency was 50 Hz during 300 km/h driving, however, the primary frequency band was between 45 and 70 Hz.

Though, if the fatigue life is not affected as the spring thickness decreases, it is considered that there will be no detriment in using a small spring thickness in a high-speed train’s VEH device. As shown in Figure 17, the resonant frequency was changed due to the driving speed of the high-speed train and the wheel–rail condition, etc. For example, the resonant frequency of the VEH device, with a spring thickness of 1.0 mm, was 50 Hz based on a speed of 300 km/h, which is the main commercial driving speed of a high-speed train. The VEH device’s power decreases due to a resonance frequency difference from the designed resonance frequency. However, as seen from the fast fourier transform (forward, FFT) analysis of Figure 17, the resonant frequency changes between 45 and 70 Hz when the high-speed train had a driving speed of around 300 km/h. Therefore, in the case of the VEH device using a spring with a thickness of 0.7 mm to set a resonant frequency of 45 Hz, the stiffness was changed, and even if the resonance frequency increases, it is possible to use the VEH device during the operation of the high-speed train.

**Figure 18:**
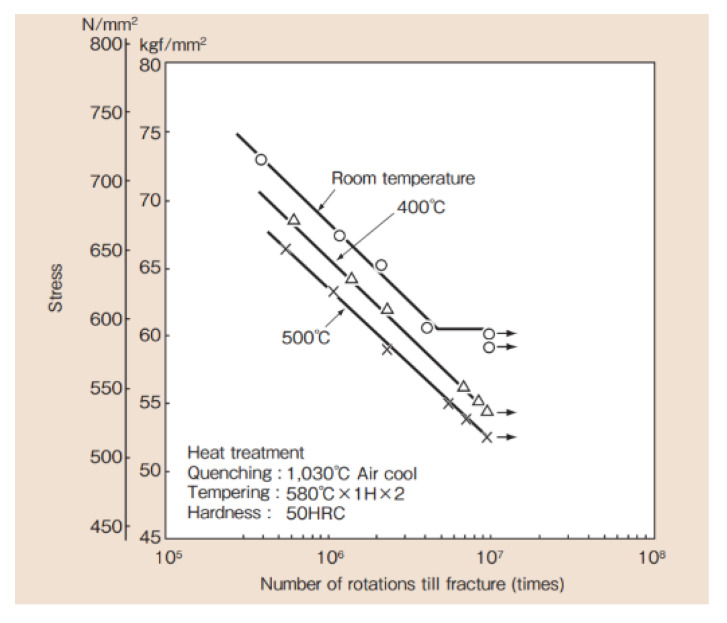
The S–N Curve obtained through the rotational bending fatigue test of the SKD 61-based material—the maximum stress of 400 MPa on the spring due to the stopper was lower than the fatigue limit of the SKD 61 material.

So, it is more important to secure the durability than change the stiffness and allow for a little change in the resonant frequency. The result of measuring the stress changed through the strain by attaching the strain gauge to the spring in Figure 16a; the maximum stress of a spring made of the SKD 61 material was confirmed to be about 400 MPa when the displacement was continuously limited to 2 mm, shown in Figure 16d, by the mechanical stopper at 2.0 G or higher. The results of the fatigue life in the S–N Curve—obtained through the rotational bending fatigue test of the SKD 61 base material, as shown in Figure 18 [49]—showed that the maximum stress of 400 MPa was lower than the fatigue limit of the SKD 61 material, and it was confirmed that durability of the VEH device using the mechanical stopper could be secured.

### 3.2. The Generation Performance Test of the Damage Prevention Design of the VEH Device Using the Mechanical Stopper in the High-Speed Train 

Many experimental studies could be found in the literature and were carried out on the amount of energy harvesting power according to the design factor of energy harvesting to verify the applicability of the energy harvesting technology that produces electric energy from a vibration energy source according to the real testbed conditions [5,40,41,42,43].

**Figure 19:**
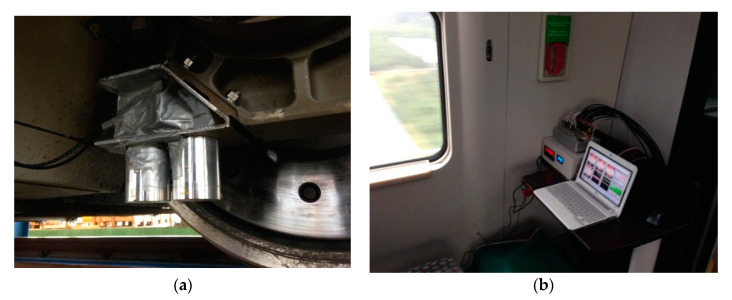
The VEH devices with the mechanical stopper for the high-speed train test: (**a**) the installation of two VEH devices on the axle box. Only the resonant frequencies were different from each other—one was 45 Hz, and the other was 50 Hz; (**b**) the voltage measurement system in the cabin.

The performance test was conducted for the VEH device with the mechanical stopper on a high-speed train test. The same VEH devices, which were confirmed through laboratory tests, were installed on the axle box of the high-speed train to verify the performance. Only the resonant frequencies were different with the VEH-1 device having a 50 Hz resonant frequency, and the VEH-2 device having a 45 Hz resonant frequency. Figure 19 shows the two VEH devices’ installation on the axle box’s test jig on the high-speed train. Each lower coil of the VEH device and the voltage measurement system in the cabin were connected to measure the generated voltage in real-time during the high-speed train drive period, as shown in Figure 19. The results were expressed in terms of one lower coil in the VEH device for the standardization of the test results above. Each coil of a VEH device can be connecting in series or parallel.

**Figure 20:**
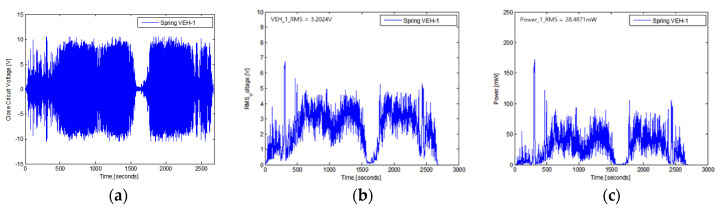
The performance test results of the VEH-1 device with the mechanical stopper, a 50 Hz resonant frequency and a 360 Ω load resistance: (**a**) the peak to peak voltage (V_pk-pk-veh-T_); (**b**) the RMS generated voltages (V_rms-veh-T_); (**c**) the experimentally generated power (P_exp-veh-T_).

**Figure 21:**
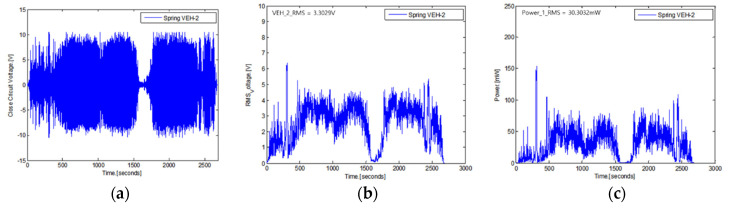
The performance test results of the VEH-2 device with the mechanical stopper, a 45 Hz resonant frequency and a 360 Ω load resistance: (**a**) the peak to peak voltage (V_pk-pk-veh-T_); (**b**) the RMS generated voltages (V_rms-veh-T_); (**c**) the experimentally generated power (P_exp-veh-T_).

The real-time generated voltage of two VEH devices with a mechanical stopper, while the high-speed train was driving at about 300 km/h, are shown in Figure 20 and Figure 21. The driving distance was 115.4 km, and this driving test included a braking test of the high-speed train for about 1500 s. The values of the peak to peak generated voltage with a 360 Ω load resistance (V_pk-pk-veh-T_) are shown in Figure 20a and Figure 21a. The maximum values of the peak to peak generated voltage with a 360 Ω load resistance were very high, measured close to ±10 V in both VEH devices. However, compared with the VEH device without the mechanical stopper, as shown in Figure 6, the maximum value of the peak to peak generated voltage with a 360 Ω load resistance was ±20 V, even when driving at a slower speed and for a shorter distance. It was a difference of about 50%. This result was consistent with limiting the spring’s moving displacement to 2 mm, 50% of the maximum moving displacement of the spring (4 mm). However, in the case of the VEH device with the mechanical stopper, it could be seen that the voltage was relatively constant at about ±10 V in the high-speed train running at a speed of 300 km/h.

The RMS generated voltages with a 360 Ω load resistance (V_rms-veh-T_) were calculated using a 1-s time interval of the peak to peak generated voltage with the load resistance at one lower coil. The V_rms-veh-T_ of the VEH-1 device was calculated as 3.3029 V, and the V_rms-veh-T_ of the VEH-2 device was calculated as 3.2024 V for a 2700-s total time interval. As the results of the V_rms-veh-T_ of the two VEH devices and Equation (3), the experimentally generated power (P_exp-veh-T_) of the VEH-1 device was calculated as 30.3032 mW for the same time interval, and the P_exp-veh-T_ of the VEH-2 device was 28.4871 mW. Therefore, the values of the total P_exp-veh-T_ of each VEH device for the sum-up of the harvested power output from the VEH’s coils (the serial connection of the upper coil and the lower coil) were calculated as 60.6064 mW and were 56.9742 mW on the high-speed train test. The difference in the total P_exp-veh-T_ was only about 6%. This confirmed that each VEH device with the mechanical stopper meets and exceeds the 50 mW threshold for the target power generation’s performance needed for the high-speed train. Especially, in the case of the VEH device using a spring with the spring thickness of 0.7 mm to set the resonant frequency of 45 Hz, the spring’s stiffness was changed, and even if the resonant frequency increases, there was no detriment in using the VEH device for the high-speed train.

Therefore, it was confirmed that the VEH device using the mechanical stopper met the design target of the power generation performance to be used as a power source of intelligent monitoring systems, such as the wireless sensor node. The durability of the VEH device could also be secured with the mechanical stopper for the high-speed train.

## 4. Results

In this study, the VEH device was designed for durability improvement. The study was carried out on the amount of energy harvesting power possible considering the design structure that prevents spring damage using a mechanical stopper to improve durability. 

The maximum impact of the vibration acceleration on the axle occurred between ± 30 and ± 50 G at a 300 km/h speed of the high-speed train. Since the VEH device uses the incoming vibration acceleration load, the vibration acceleration has the characteristic of being an energy source and an external force that influences the durability. Therefore, the spring’s metal material of the VEH device does not easily have durability when high vibration accelerations are inputted in the form of an impact load and periodic. First, the finite element analysis (FEA) of the frequency response for the VEH device was conducted comparing the maximum displacement and the maximum stress of the spring according to the vibration acceleration. The displacement of about 4.0 mm was observed at a 2.0 G vibration acceleration. It was confirmed that the maximum stress value was very high at about 2000 MPa, and it was 3000 MPa at 3.0 G.

The experiment in the laboratory confirmed a similar result of the finite element analysis (FEA). The maximum value of the moving displacement for the spring was about 4.0 mm at 2.0 G. If the values of the generated voltage were over 4.25 V, the value of generated power (P_exp-veh_) in the VEH device would be over 50 mW, which is the minimum target performance of the VEH device for the train systems. The minimum value of the moving displacement of the spring (from 0 mm to the upside) was about 1.3 mm at 0.5 G to surpass 4.25 V. Additionally, the moving displacement of the spring was determined to be as 2.0 mm with the mechanical stopper. However, the displacement limits also reduced the generated voltage (V_rms-veh_), resulting in a value of the generated voltage (V_rms-veh_) of up to 9.41 V at a 3.0 G vibration acceleration, at which the maximum value of the generated power (P_exp-veh_) was calculated at 245.97 mW in the laboratory.

A study was also conducted to find the effects of changing the displacement of the spring and the stress change according to spring thickness under the limits of the moving displacement, a spring thickness of 0.7 mm, and a 45 Hz resonant frequency in the 0.3 to 3.0 G range of the vibration acceleration. The moving displacement of the spring was limited to 2.0 mm for an above 2.0 G vibration acceleration by applying a mechanical stopper. However, the resonance frequency significantly changed, increasing the vibration acceleration. The result of measuring the stress change through the strain by attaching a strain gauge to the spring showed that the maximum stress of spring made of SKD 61 material was around 400 MPa when the displacement is continuously limited to 2 mm by the mechanical stopper at 2.0 G or higher. The maximum stress of 400 MPa was lower than the fatigue limit of SKD 61 in the S–N curve, and it was confirmed that the durability of the VEH device using the mechanical stopper could be secured.

Finally, the performance test was conducted for the VEH device with a mechanical stopper through the high-speed train test. The voltage was relatively constant at around ±10 V in the high-speed train running at a speed of 300 km/h. The experimental values of the total P_exp-veh-T_ of each VEH device with the mechanical stopper were 60.6064 and 56.9742 mW, measured on the high-speed train test. It confirmed that each VEH device with the mechanical stopper meets and exceeds the 50 mW threshold of the design target of the power generation’s performance for the train system, and the durability could be secured for the high-speed train.

## Figures and Tables

**Table 1:** The maximum values of the generated power with a 360 Ω load resistance (P_exp-veh_) at one lower coil of the VEH device without the mechanical stopper. The maximum moving displacement of the spring was 4.0 mm.

Vibrating Acceleration (G)	Vibration Displacement (mm)	Maximum RMS Voltage, V_rms-veh_ (V)	Maximum Generated Power, P_exp-veh_ (mW)
0.5	2.18	9.78	265.69
1.0	3.25	15.11	634.20
2.0	3.81	16.60	765.44

**Table 2:** The maximum values of the generated power with a 360 Ω load resistance (P_exp-veh_) at one lower coil of the VEH device with the mechanical stopper. The maximum moving displacement of the spring was 2.0 mm.

Vibrating Acceleration (G)	Vibration Displacement (mm)	Maximum RMS Voltage, V_rms-veh_ (V)	Maximum Generated Power, P_exp-veh_ (mW)
0.5	1.88	8.01	178.22
1.0	2.05	8.53	202.11
2.0	2.21	9.02	226.00
3.0	2.34	9.41	245.97
